# Prospective Epidemiological Research on Functioning Outcomes Related to Major Depressive Disorder in Japan (PERFORM-J): Protocol for a Prospective Cohort Study

**DOI:** 10.2196/resprot.9682

**Published:** 2018-06-25

**Authors:** Tomiki Sumiyoshi, Koichiro Watanabe, Shinichi Noto, Shigeru Sakamoto, Yoshiya Moriguchi, Shuichi Okamoto

**Affiliations:** ^1^ Department of Preventive Intervention for Psychiatric Disorders National Institute of Mental Health National Center of Neurology and Psychiatry Tokyo Japan; ^2^ Department of Clinical Epidemiology Translational Medical Center National Center of Neurology and Psychiatry Tokyo Japan; ^3^ Department of Neuropsychiatry Kyorin University School of Medicine Tokyo Japan; ^4^ Department of Health Sciences Niigata University of Health and Welfare Niigata Japan; ^5^ Japan Medical Affairs, Japan Pharma Business Unit Takeda Pharmaceutical Company Limited Tokyo Japan; ^6^ Medical Affairs Lundbeck Japan KK Tokyo Japan

**Keywords:** antidepressants, depression, major depressive disorder, cognitive dysfunction, observational study

## Abstract

**Background:**

Patients with major depressive disorder may exhibit cognitive dysfunction that can affect functional outcomes. However, the prevalence and burden of cognitive dysfunction in Japanese patients with MDD have not been thoroughly examined.

**Objective:**

To investigate the time course (over 6 months) of several functional outcomes during treatment with antidepressants in Japanese patients with major depressive disorder. The primary objective is to assess longitudinal changes in cognitive function and depressive symptoms, using both clinician-rated and patient-rated scales. The study incorporates assessments of cognitive function and other functional outcomes (functional capacity, disability, work productivity and impairments of activity, and quality of life), as well as depressive symptoms.

**Methods:**

PERFORM-J (Prospective Epidemiological Research on Functioning Outcomes Related to Major Depressive Disorder in Japan) is a 6-month, prospective, multi-center, epidemiological cohort study. Participants are Japanese outpatients aged 18-65 years with a recurrent or new diagnosis of a major depressive episode (according to the Diagnostic and Statistical Manual of Mental Disorders, 4th Edition, Text Revision [DSM-IV-TR]), who are initiating a new antidepressant as monotherapy (either as ﬁrst-line therapy or after switching from a previous antidepressant). Eligible patients are evaluated objectively during four visits (at baseline and at Months 1, 2, and 6) using physician-rated assessments of severity of depressive symptoms, cognitive function, and functional capacity. Subjective, patient-reported, outcomes are also assessed as indicators of depressive symptoms, disability, work productivity or impairments of activity, and perceived cognitive dysfunction.

**Results:**

The study began in September 2016. Patient enrollment was completed on June 30, 2017, with 523 patients having been enrolled from 48 study sites. As of October, 2017, 279 patients had completed the study.

**Conclusions:**

PERFORM-J is expected to provide valuable information on the longitudinal relationship between cognitive dysfunction, depressive symptoms, and other functional outcomes in Japanese patients with major depressive disorder who initiate monotherapy with antidepressants.

**Trial Registration:**

UMIN Clinical Trials Registry UMIN000024320; https://upload.umin.ac.jp/cgi-open-bin/ctr_e/ctr_view.cgi? recptno=R000028011 (Archived by WebCite at http://www.webcitation.org/70K7W9PgC).

**Registered Report Identifier:**

RR1-10.2196/9682

## Introduction

Major depressive disorder (MDD) is a common psychiatric condition, with an estimated 12-month prevalence of 2.2% in Japan [1,2]. Although mood disturbances are characteristic of patients with MDD, there is accumulating evidence for the presence of cognitive dysfunction, linked with poor functional outcomes [3-5]. For example, in a recent large-scale, cross-sectional study conducted in six Asian countries and territories, 67% of currently depressed, nonmedicated outpatients reported subjective memory deficits and 73% experienced concentration deficits [6]. In addition, Japanese treatment guidelines for depression recommend that cognitive function should be carefully monitored, even after remission of depressive (affective) symptoms has been achieved [7]. This is partly due to a possibility of relapse of affective symptoms (attributable to the continued presence of cognitive dysfunction) once patients return to normal daily activities. Little is known, however, about longitudinal changes in cognitive function throughout depressive episodes and periods of remission, or the impact of cognitive dysfunction on symptomatic remission and functional recovery in patients with MDD.

PERFORM (Prospective Epidemiological Research on Functioning Outcomes Related to Major Depressive Disorder; NCT01427439) was a 2-year, prospective, noninterventional cohort study of European patients with MDD, conducted in real-world settings in France, the UK, Spain, Germany, and Sweden [8,9]. The study aimed to investigate changes in cognitive function and functional outcomes during depressive episodes. The findings suggest that perceived cognitive dysfunction is linked with poor overall functioning, including reductions in work productivity and impairment in quality of life. Moreover, the relationships between cognition and functional outcomes were not fully explained by changes in severity of depressive symptoms [8]. Additionally, depressive symptoms and disturbances in self-perceived cognition followed different trajectories during treatment with antidepressants; improvements in cognitive function were more gradual than improvements in mood symptoms, with a lesser magnitude [8].

The same investigators reported a significant association between residual subjective cognitive deficit (assessed using the five-item Perceived Deficit Questionnaire [PDQ-5] [10]) and subsequent relapse (assessed 6 months later) in MDD patients whose symptoms had remitted during treatment for 2 months [9]. In fact, the odds of relapse increased by 12% with each unit increase in PDQ-5 score. Based on these observations, the authors concluded that residual mood symptoms may identify patients at risk of relapse, and that interventions to reduce residual cognitive dysfunction may potentially reduce the risk of relapse of depressive symptoms [9]. Due to regulations around noninterventional studies in Europe, PERFORM employed patient-rated scales but limited application of physician-rated measures. However, reports by patients do not always correlate well with physician-rated outcomes [11,12].

Results from the World Mental Health Japan Survey suggest that the male to female ratios in both prevalence and persistence of depression in Japan are different from those reported in Western countries: prevalence is higher in middle-aged men, while persistence is higher among women and younger groups [1]. Furthermore, treatment rates in Japan are lower than those in high-income Western countries, which may be partially explained by less health service expenditure, [13] and partially by the stigma associated with mental illnesses in Japan [1]. Such differences suggest that the findings of the PERFORM study may not be extrapolated to Japanese patients and warrant further investigation. To our knowledge, no studies have explored longitudinal changes in cognitive function and higher-level functional outcomes during treatment of Japanese patients with MDD [14]. To increase awareness of cognitive dysfunction in depression in Japan, we have designed a prospective observational study (PERFORM-J; UMIN Clinical Trials Registry: UMIN000024320), based on the original PERFORM study. Since patient-rated and physician-rated measures seem to represent qualitatively different aspects of cognition and psychosocial function, both tools are used in PERFORM-J to explore longitudinal changes in cognitive function and other aspects of functional outcomes in Japanese patients with MDD.

## Methods

### Study Objectives

The primary objective of PERFORM-J is to examine longitudinal changes in cognitive function and depressive symptoms in patients with MDD over a period of 6 months, following the start of treatment with an antidepressant in routine clinical practice. Secondary objectives are (1) to determine the number of patients with cognitive dysfunction and/or depressive symptoms across the study period; (2) to examine the relationship between cognitive disturbance and psychosocial function and work productivity or activity impairment; and (3) to compare quality of life and utilization of healthcare resources longitudinally, between patients with different severities of cognitive dysfunction. Additional exploratory objectives are (1) to determine whether the presence of cognitive dysfunction is a risk factor for treatment refractory depressive symptoms at month 1, failure to achieve symptomatic remission at month 2 or 6, and relapse of depressive symptoms at month 6; and (2) to investigate the association between cognitive dysfunction and functional capacity.

### Patients

Eligible participants are Japanese outpatients aged 18-65 years with a recurrent or new diagnosis of a major depressive episode (as defined by the Diagnostic and Statistical Manual of Mental Disorders, 4th Edition, Text Revision [DSM-IV-TR]), who initiate a new antidepressant as monotherapy (a tricyclic or tetracyclic antidepressant, selective serotonin reuptake inhibitor, serotonin–norepinephrine reuptake inhibitor, or noradrenergic and/or serotonergic antidepressant) either as ﬁrst-line therapy or switching from a previous antidepressant, based on the judgement of their physician. The baseline visit is defined as a clinic visit at which the new antidepressant is initiated. The full eligibility criteria are shown in Textbox 1. Eligible patients are enrolled during the first visit (visit 1). As the study is conducted during routine clinical practice, following enrollment (on initiation of a new antidepressant as monotherapy) subsequent addition of other antidepressants (and subsequent dose modification) is permitted during the study period, and use of other concomitant medications is permitted. The dosage and administration of antidepressants are in accordance with general prescribing instructions.

### Assessments

During the baseline visit, informed consent is obtained, and eligibility to participate in the study is determined (according to the criteria in Textbox 1). Demographic data are then collected, before the new antidepressant is prescribed, as detailed in Table 1. Information on the management of MDD and utilization of healthcare resources is also collected at each subsequent visit.

**Inclusion and exclusion criteria.**

**Inclusion criteria**

1. An outpatient.
2. Aged between 18 and 65 years at the time of giving informed consent.
3. With a recurrent or new diagnosis of a major depressive episode according to the Diagnostic and Statistical Manual of Mental Disorders-IV (DSM-IV-TR); the diagnosis is confirmed using the Major Depressive Episode module of the Mini International Psychiatric Interview (MINI) [15].
4. Initiating a new antidepressant as monotherapy (a tricyclic or tetracyclic antidepressant, a selective serotonin reuptake inhibitor, a serotonin–norepinephrine reuptake inhibitor, or a noradrenergic and specific serotonergic antidepressant) at the baseline visit (either as ﬁrst-line therapy or after switching from a previous antidepressant), as decided by the investigator.
5. Capable of understanding the content of the clinical research and complying with the research protocol requirements (in the opinion of the investigator)
6. Capable of signing and dating an informed consent form, before initiation of the clinical research procedures.
7. Capable of reading and understanding the research questionnaires.

**Exclusion criteria**

1. A concurrent diagnosis or history of any of the following conditions:
  - schizophrenia or other psychotic disorders
  - bipolar disorder
  - dementia, or any other neurodegenerative disease
  - substance dependence, including dependence on alcohol and other drugs, but not including mild and moderate nicotine dependence; patients with severe nicotine dependence are excluded
  - any psychiatric disorder due to a general medical condition or substance misuse.
2. A prescription for more than one antidepressant on the day of the baseline visit (for example, a combination of two or more antidepressants).
3. An antipsychotic prescription on the day of the baseline visit.
4. A prescription for a mood stabilizer on the day of the baseline visit.
5. Current treatment with electroconvulsive therapy or repeated transcranial magnetic stimulation.
6. Pregnant or breastfeeding (female patients) at the beginning of the study.
7. Acute suicidality (in the opinion of the investigator).
8. Currently enrolled in an interventional clinical research study, such as a clinical trial.
9. A workmate of the investigator or his/her immediate family, or a subordinate of the investigator or their immediate family.
10. Prior enrolment in the study.
11. Unlikely to comply with the protocol (in the opinion of the investigator).

**Table 1:** Data collection (DSM-IV-TR: Diagnostic and Statistical Manual of Mental Disorders-IV; MDD: Major Depressive Disorder; VAS: visual-analogue scores).

Data to be collected			Details
Demographic information			Age; gender; weight; height; use of tobacco; marital status; living area; educational level; employment status
MDD history			Number of depressive episodes; timing and treatment of previous depressive episode; history of suicide attempts and hospitalizations for depression; concomitant mental and somatic conditions (including diagnosis and treatment, if relevant)
Management of current episode of MDD			Antidepressant treatments prescribed; concomitant psychotropic treatments; use of psychotherapy
Utilization of healthcare resources			Number of visits to physicians or other health care professionals; hospitalizations (duration; ward; relationship to depression) and absences from employment due to illness (duration and relationship to depression) due to any problems (not limited to MDD symptoms)
Life events			Occurrence of critical life events (eg, death of a family member/relative; unemployment, relocation)
**Physician-rated assessments**			
	Mini International Neuropsychiatric Interview (MINI) [15]	Brief structured diagnostic interview for major Axis I psychiatric disorders in DSM-IV-TR. Diagnosis of major depressive episode confirmed using “Major Depressive Episode” module	
	Clinical Global Impression - Severity (CGI-S) scale [17]	Patient's current state of mental illness, according to the physician’s experience. 7-point scale from 1 (normal; not at all ill;) to 7 (among the most extremely ill patients)	
	Montgomery-Asberg Depression Rating Scale (MADRS) [18]	Severity of depressive episodes. 10 items rated from 0 (normal findings or absence of symptoms) to 6 (severe depressive symptoms). Total score=0–60 (higher score=more severe depression). Remission indicated by score ≤10	
	Digit Symbol Substitution Test (DSST) [16,25]	Speed of psychomotor performance (visual perception, spatial decision-making, and motor skills). The task is to match 133 digits with simple symbols in 120 seconds. Correct answers are counted; score=0–133	
	University of California, San Diego, Performance-based Skills Assessment-Brief (UPSA-B)^a^ [19,26]	Functional skills in patients with mental illness (brief version of the role-play-based performance test battery). Two subscales (managing finances and communication with others); raw scores are rescaled to a range of 0 to 100. Higher score=greater functional capability	
**Patient-reported outcomes**			
	Patient Health Questionnaire-9 item (PHQ-9) [20,27]	Depressive symptoms. 9 items rated from 0 (not all) to 3 (nearly every day). Total score=0 (absence of depression) to 27 (severe depression)	
	Sheehan Disability Scale (SDS) [21]	Disability assessed in three domains (work/school; social life/leisure activities; family life/home duties). Three discretized 10-point VAS from 0 (no disability) to 10 (extreme disability). The sum of scores provides a single measure of global functional impairment: range=0 (unimpaired) to 30 (highly impaired)	
	Work Productivity and Activity Impairment (WPAI) questionnaire [22]	Work productivity/impairment in activity in past 7 days (6 items). Yields scores for: absenteeism; presenteeism; loss of work productivity; and impairment of activity. Outcomes are expressed as % impairment. Higher scores=worse outcomes	
	Perceived Deficits Questionnaire-Depression (PDQ-D) [23]	Perceived cognitive deficits in MDD patients. 20 items in 4 subscales: attention/concentration; retrospective memory; prospective memory: planning/organisation. Score=0 (never in past 7 days) to 4 (very often, more than once a day). Total score=sum of raw scores; range=0 to 80. High score=poorly perceived cognitive dysfunction	
	EuroQol 5-Dimensions, 5-Levels Questionnaire (EQ-5D-5L) [24]	Generic health status (preference-based measure of well-being). Five items: mobility, self-care, usual activities, pain/discomfort, depression/anxiety, plus VAS for overall health state. Used to calculate a utility index: range=0 (death) to 1 (perfect health). VAS scores range from 0 (worst health state) to 100 (best health state)	

^a^To be performed only in patients from preselected research sites (approximately 100 patients).

At the beginning of the study (baseline; visit 1), month 1 (visit 2), month 2 (visit 3), and month 6 (visit 4), severity of the illness, depressive symptoms, and cognitive function are assessed using the Clinical Global Impressions-Severity (CGI-S) scale, the Montgomery-Asberg Depression Rating Scale (MADRS), and the Digit Symbol Substitution Test (DSST), respectively [16-18]. Functional capacity is evaluated for a subset of approximately 100 patients at selected sites using the University of California, San Diego Performance-based Skills Assessment-Brief (UPSA-B) [19]. Additionally, all patients are asked to complete the following self-assessment questionnaires to evaluate subjective depressive symptoms, cognition, social function, and quality of life: the Patient Health Questionnaire-9 item (PHQ-9) [20]; Sheehan Disability Scale (SDS) [21]; Work Productivity and Activity Impairment questionnaire (WPAI) [22]; Perceived Deficits Questionnaire-Depression (PDQ-D) [23]; and EuroQol5-Dimension, 5-Level (EQ-5D-5L) [24]. Details of all physician-rated and self-reported assessment tools are provided in Table 1. Validated Japanese versions of these assessment tools are used [25-27]. The same instruments are administered at each visit throughout the course of the study (Table 2). The investigators will inform the marketing authorization holders of each drug if suspected adverse events occur.

The study is conducted in accordance with the ethical principles described in the Declaration of Helsinki and the Japanese Ethical Guideline for Clinical Research, as well as all other applicable laws and regulations. Ethical review committees are constituted according to the regulations and approve the study protocol at each site before commencement of the study. Patients are required to provide written informed consent and are free to withdraw from the study at any time.

### Statistical Analysis

The analysis population will comprise all eligible patients who complete at least one DSST assessment during the study period. Analyses will be based on observed cases, with no imputation of missing data.

For the primary analyses, changes from baseline at each assessment time point will be calculated for DSST and MADRS scores. Individual DSST scores will be allocated to one of four categories, as follows: within norm; 0.33-0.67 SD below norm; 0.67-1 SD below norm; or ≥1 SD below norm. Separately, DSST scores will be allocated to dichotomous (yes/no) categories as follows: within norm; ≥0.33 SD below norm; ≥0.67 SD below norm; ≥1 SD below norm. All cutoff values will be adjusted for the patient’s age at baseline, as described in the Wechsler Adult Intelligent Scale - Third Edition (Japanese translation) [25]. MADRS scores will be categorized as none, mild, moderate or severe, as previously described [28,29].

**Table 2:** Schedule of assessments (CGI-S: Clinical Global Impression-Severity; DSST: Digit Symbol Substitution Test; EQ-5D-5L: EuroQoL 5-Dimensions, 5-levels Questionnaire; MADRS: Montgomery-Asberg Depression Rating Scale; MDD: Major Depressive Disorder; MINI: Mini International Neuropsychiatric Interview; PDQ-D: Perceived Deficits Questionnaire-Depression; PHQ-9: Patient Health Questionnaire-9 item; SDS: Sheehan Disability Scale; UPSA-B: University of California, San Diego Performance-based Skills Assessment-Brief; WPAI: Work Productivity and Activity Impairment questionnaire).

Study visit			1		2		3		4
Approximate time of visit, months			Baseline		Month 1		Month 2		Month 6
Approximate time of visit, days			Day 1		Day 29		Day 57		Day 169
Allowance, days^a^			1		22 to 36		43 to 71		141 to 197
Patient information/consent			X^b^						
Demographics			X						
MDD history			X						
Occurrence of life events			X						
MDD management			X		X		X		X
Healthcare resource use			X		X		X		X
**Physician-rated assessments**									
	CGI-S	X		X		X		X	
	MINI	X							
	MADRS	X		X		X		X	
	DSST	X				X		X	
	UPSA-B^c^	X				X		X	
**Patient-reported outcomes**									
	PHQ-9	X		X		X		X	
	SDS	X		X		X		X	
	WPAI	X		X		X		X	
	PDQ-D	X		X		X		X	
	EQ-5D-5L	X		X		X		X	

^a^The starting date of antidepressant treatment (ie, the first day of administration) is defined as Day 1.

^b^Informed consent is required before any clinical research procedures are performed.

^c^Conducted only in patients at preselected research sites (approximately 100 patients).

For the secondary analyses, changes from baseline at each assessment time point will be calculated for SDS, WPAI, UPSA-B, PDQ-D and EQ-5D-5L scores. The association between cognitive function and depressive symptoms will be assessed using the DSST and MADRS categories at each visit. Correlations between DSST scores and SDS, WPAI, and EQ-5D-5L scores (individually) will be calculated using Pearson's and Spearman's correlation coefﬁcients. SDS, WPAI, UPSA-B, and EQ-5D-5L scores at each assessment point will be compared using analysis of variance models, according to the DSST category at baseline. Similar analysis will be performed for PDQ-D as the primary measure of cognitive function (scores will be allocated to one of four categories; the cutoffs being based on quartiles).

As an exploratory investigation, univariate and multivariate logistic regression analyses will be conducted to determine whether the presence of cognitive dysfunction is a predictor of response, remission, and relapse of depressive symptoms. Patients with a ≥50% reduction from baseline in MADRS score at month 1 are categorized as treatment responders. Patients with a MADRS score of ≤10 at month 2 and/or 6 are categorized as having achieved symptomatic remission. Patients who achieved remission at an earlier visit and have a MADRS score ≥22 at month 6 are categorized as having relapsed in terms of depressive symptoms. Factors for the logistic regression analyses (eg, DSST score, PDQ-D score, MADRS score, age, gender, educational level, and employment status), will be selected for their clinical relevance based on the literature [9]. Exploratory analyses will also evaluate correlations between scores on the UPSA-B, DSST, WPAI (if the sample size allows), EQ-5D-5L, and MADRS scales. Statistical tests will be two-sided at the 5% signiﬁcance level, if not otherwise specified.

In subgroup analyses, all data will be summarized according to the MADRS category at baseline, and according to age group.

A target sample size of 500 patients was estimated in order to ensure acceptable precision of the mean change from baseline in DSST scores at months 2 and 6. Based on two previous studies which investigated the effect of vortioxetine on cognitive function in MDD [30,31], assuming a standard deviation of 8.1 and a withdrawal rate of 25% at month 6, 500 enrolled patients will ensure a value of 0.82 for the half-width of the two-sided 95% confidence interval for the change in DSST score from baseline to month 6.

## Results

The PERFORM-J study began in September 2016. Patient enrollment was completed on June 30, 2017, with 523 patients having been enrolled from 48 sites, with the UPSA-B assessment having been conducted on 141 patients. As of October 2017, 279 patients have completed the study.

## Discussion

PERFORM-J is the first study conducted in Japan to prospectively evaluate longitudinal changes in cognitive function, depressive symptoms, and other functional outcomes in patients with MDD following the start of antidepressant therapy. The information obtained will shape our understanding of how antidepressants affect cognition in Japanese patients. Importantly, the study includes both physician- and patient-rated tests of cognitive function, administered to a large group of patients treated in accordance with current clinical practice in Japan. A noninterventional design was chosen to avoid interference with standard practice.

The prospective design and the number of study sites should facilitate collection of data that is sufficient to represent the MDD population in Japan. The 6-month study period should be long enough to detect the effects of antidepressants on depressive symptoms. The study period will also allow detection of meaningful changes in functional outcomes and utilization of healthcare resources over time. In fact, the study design is similar to that of PROACT (Prospective Research Observation to Assess Cognition in Treated MDD patients), currently ongoing in China, and PERFORM-K in South Korea [32].

Currently, cognitive function does not appear to be a major concern among Japanese psychiatrists who treat mood disorders. Therefore, the current study should provide valuable information to facilitate improvements in functional outcomes in patients with MDD.

A major limitation of this study relates to the uncontrolled and observational study design, which does not allow evaluation of cause and effect relationships. Additionally, patients can take concomitant medication, potential effects of which on cognition cannot be excluded.

In conclusion, we have described the protocol of a prospective, multi-center study of the longitudinal relationship between cognitive dysfunction, severity of depressive (mood) symptoms, and higher-level functional outcomes in Japanese patients with MDD. Results from PERFORM-J should provide valuable information regarding the potential of antidepressant monotherapy to improve symptoms and social functioning in such patients.

## Abbreviations

CGI-S: Clinical Global Impression - Severity
DSM-IV-TR: Diagnostic and Statistical Manual of Mental Disorders, 4th Edition, Text Revision
DSST: Digit Symbol Substitution Test
EQ-5D-5L: EuroQoL 5-Dimensions, 5-levels
MADRS: Montgomery-Asberg Depression Rating Scale
MDD: Major Depressive Disorder
MINI: Mini International Neuropsychiatric Interview
PDQ-5: Perceived Deficit Questionnaire-5 item
PDQ-D: Perceived Deficits Questionnaire-Depression
PHQ-9: Patient Health Questionnaire-9 item
SDS: Sheehan Disability Scale
UPSA-B: University of California, San Diego Performance-based Skills Assessment-Brief
VAS: Visual Analog Scale
WPAI: Work Productivity and Activity Impairment questionnaire
